# Sphingosine-1-phosphate in anti-neutrophil cytoplasmic antibody-associated vasculitis: coagulation-related clinical indicators and complications

**DOI:** 10.1042/BSR20200157

**Published:** 2020-10-30

**Authors:** Kai-Li Wu, Qing-Hui Liang, Na Ding, Bo-Wei Li, Jian Hao

**Affiliations:** 1 Renal Division, Department of Medicine, The Affiliated Hospital of Inner Mongolia Medical University, Huhehot, Inner Mongolia 010050, China; 2 Inner Mongolia Medical University, Huhehot, Inner Mongolia 010059, China

**Keywords:** S1P, ANCA, vasculitis, D-dimer, coagulation complications

## Abstract

**Background:** Sphingosine-1-phosphate (S1P) plays a significant role in anti-neutrophil cytoplasmic antibody (ANCA)-associated vasculitis (AAV).

**Methods:** We collected the plasma samples from 40 patients with AAV and 10 healthy volunteers. The plasma levels of S1P were tested by enzyme-linked immunosorbent assay (ELISA). The levels of serum creatinine (Scr) were tested by rate method, and then the estimated glomerular filtration rate (eGFR) of the patients was calculated from the Scr, age, and gender. Prothrombin time (PT), partial thromboplastin time (APTT), thrombin time (TT), fibrinogen (FIB), fibrinogen reduction product (FDP), D-dimer and C-reactive protein (CRP) were tested by turbidimetric inhibition immunoassays. Platelets (PLTs) were tested by fluorescently labeled electrical impedance method.

**Results:** The plasma levels of S1P were significantly higher in AAV patients than in healthy volunteers. Correlation analysis showed that plasma levels of S1P were negatively correlated with glomerular filtration (*P*=0.022, *r* = −0.306), and positively correlated with circulating levels of Birmingham vasculitis activity score (BVAS), PLT and D-dimer, (*P*=0.004, *r* = 0.443; *P*<0.001, *r* = 0.654; *P*=0.006, *r* = 0.427). The 40 patients with AAV were classified into three groups: the thromboembolism group (with complications of cerebral infarction and myocardial infarction, *n*=6), cerebral ischemia group (*n*=4), and cerebral hemorrhage group (*n*=2). The plasma levels of S1P were highest in the thromboembolism group and lowest in the cerebral hemorrhage group (*P*=0.003).

**Conclusions:** Plasma levels of S1P were associated with circulating levels of D-dimer, PLT and BVAS in the patients with AAV. Hence, plasma S1P level can be used as a biomarker to predict coagulation-related complications in AAV.

## Introduction

Anti-neutrophil cytoplasmic antibody (ANCA)-associated vasculitis (AAV) is a type of necrotizing vasculitis, with an annual incidence of approximately 10–20 per million [1]. It has three main clinical types, granulomatous with polyangiitis (GPA), microscopic polyangiitis (MPA) and eosinophilic granulomatous polyangiitis (EGPA) [2], in which serum markers include protease 3 (PR3) and myeloperoxidase (MPO) [3,4] that are released under the stimulation of pathogens [5–8].

Sphingosine-1-phosphate (S1P) is a sphingolipid metabolite that regulates inflammation and immune responses [9,10]. It can bind to cell-specific receptors, and plays a role in cell growth, proliferation, angiogenesis, inhibition of apoptosis and lymphocyte migration [11]. It is a ligand for a family of five G protein-coupled receptors (S1PR1–5) [12,13]. The roles of S1P and S1PR in autoimmune diseases are current research hotspots.

In systemic lupus erythematosus (SLE), S1PR modulators can prevent the migration of lymphocytes from the lymphoid organs through the antagonistic function of the S1PR. Therefore, S1PR can be a therapeutic target for SLE [14]. In sickle cell disease [13,15], rheumatoid arthritis, asthma, multiple sclerosis and inflammatory bowel disease, biologically active S1P is an essential mediator of the inflammatory immune response. S1P formation is catalyzed by sphingosine kinase (SK), and tumor necrosis factor-α (TNF-α) can activate SK1 isoenzymes. SK1 is required to mediate the TNF-α inflammatory response in cells [16].

S1P is involved in renal autoimmune diseases. In IgA nephropathy patients, the plasma S1P levels decrease, and urine S1P levels increase [17]. In anti-basement membrane antibody glomerulonephritis (GBM), the S1P receptor modulator FTY720 can prolong the survival time of patients, improve patients’ proteinuria and serum creatinine (Scr) levels, and reduce GBM [18]. In diabetic nephropathy, inhibiting the level of S1P will reduce the damage of renal tubular interstitial cells [19]. Plasma S1P levels are increased in AAV disease [9,20].

Although the pathogenesis of AAV remains unclear, previous studies have found that the complement system plays a vital role in the pathogenesis [4,9,20,21]. Among the members of the complement system, C5a is the most crucial complement, which can be activated via the three pathways [9,18,22,23]. Hao et al. found that C5a and its receptors on neutrophils can stimulate the development of AAV disease *in vivo* and *in vitro* [24], and C5a triggered S1P can further activate neutrophils. Interaction between C5a and S1P is involved in ANCA-mediated neutrophil respiratory degranulation and burst [9,25].

Recent studies have shown that the interaction between C5a and the plasma levels of S1P stimulates neutrophils and produces thrombin [26], which can destroy the integrity of endothelial cells during the coagulation process, and generate new S1P to enhance endothelial cell disruption, which is detrimental to endothelial cell remodeling [20,27,28]. This may explain why AAV patients are in a hypercoagulable state [29,30], with higher thromboembolisms and cardiovascular and cerebrovascular events than healthy volunteers [27].

Therefore, we hypothesized that plasma S1P levels are associated with coagulation-related clinical indicators, complications and disease activity in the patients with AAV.

## Methods

### Blood samples of patients

Blood samples were taken from 40 patients with AAV at the Affiliated Hospital of Inner Mongolia Medical University between December 2018 and October 2019. These patients were diagnosed by the Nephrology Department of Inner Mongolia Medical University, based on the Chapel Hill Consensus Conference of AAV naming [4]. Patients with autoimmune diseases, such as IgA nephropathy, lupus nephritis (LN), rheumatoid arthritis and Graves’ disease were excluded, as well as those with secondary vasculitis, comorbid renal disorders, or any infection. We collected the plasma from 5 ml fasting blood samples in the morning, by centrifuging at 3000×***g*** at 4°C within 5 min. The plasma samples were stored at −20°C till use, and repeated freeze–thaw cycles were avoided. Disease activity in AAV was based on the Birmingham vasculitis activity score (BVAS) [31]. Written informed consent was obtained from the 40 AAV patients and 10 healthy volunteers before taking their blood samples. This research was in compliance with the Helsinki Declaration, and was approved by the Ethics Committee of Inner Mongolia Medical University.

### Detection of S1P by enzyme-linked immunosorbent assay

Plasma levels of S1P were tested by enzyme-linked immunosorbent assay (ELISA) using a commercial kit (Echelon, Utah, U.S.A.). The 96-well microtiter plate was coated with S1P and blocked to reduce non-specific binding. S1P standards and samples were then mixed with anti-S1P antibodies, and the mixture was added to the S1P-coated plates. Antibodies competing for binding to S1P bound to the plate or sample. After incubating and washing the plate, streptavidin-horseradish peroxidase (HRP) was added to the plate, which attached to all anti-S1P antibodies (labeled with biotin) bound to the plate. After additional incubation and washing, tetramethylbenzidine (TMB) substrate was added to the plate, and the reaction was stopped by adding sulfuric acid. The absorbance was measured at 450 nm, with 630 nm as the reference wavelength. The concentration of S1P in the sample was determined by comparison with the standard curve [32].

### Detection of prothrombin time, partial thromboplastin time, thrombin time, fibrinogen, fibrinogen reduction product, Scr, C-reactive protein, platelet and D-dimer

Prothrombin time (PT) [33], partial thromboplastin time (APTT) [34], thrombin time (TT) [35], fibrinogen (FIB) [36] and fibrinogen reduction product (FDP) were determined by immunoturbidimetry using commercial kits from SIEMENS (Berlin, Germany) [37], Scr was determined by immunoturbidimetry using a commercial kit from Beckman (CA, U.S.A.) [38], C-reactive protein (CRP) was determined by immunoturbidimetry using a commercial kit from Goldsite (Shenzhen, China) [39], platelet (PLT) [40] and D-dimer [41] were also determined by immunoturbidimetry using commercial kits from SIEMENS (Berlin, Germany). Based on Scr, age and gender, the estimated glomerular filtration rate (eGFR) of the patient was calculated. The formula is the MDRD formula adapted by Chinese experts for the Chinese population.

The eGFR calculation formula for females was eGFR (ml/min/1.73 m^2^) = 186 × Scr − 1.154 × (age) − 0.203 × 0.742, × 1.227, and for males: eGFR (ml/min/1.73 m^2^) = 186 × Scr − 1.154 × (age) − 0.203 × 1.227 (Ma, [42].

### Statistical analysis

Data analysis was conducted using SPSS19.0 software package. The Student’s *t* test was used to analyze the differences in quantitative parameters between the two groups of standard distribution data. The s-test was used to analyze the differences in quantitative parameters between the three groups of standard distribution data, and represented as mean ± standard deviation (SD) as descriptive statistics. The Shapiro–Wilk test, median and interquartile range (IQR) were used to examine whether the data were normally distributed. If the two sets of data did not conform to the normal distribution and homogeneity of variance, the Spearman’s rank correlation was used to perform a correlation analysis between two continuous variables. If the two sets of data conformed to the normal distribution and homogeneity of variance, the Pearson’s rank correlation was used to perform a correlation analysis between two continuous variables. The difference was considered statistically significant if *P*<0.05.

## Results

### General data of the AAV patients and healthy volunteers

Of the 40 patients with AAV, 19 (47.5%) were males and 21 (52.5%) were females. The average age at diagnosis was 57.7 ± 10.7 (range 48.0–78.0) years. Among the healthy volunteers, five were males and five were females, with the average age of 56.1 ± 12.2 (range 22.0–78.0) years. There were no significant differences in gender (*P*=0.833) and age (*P*=0.677) between the AAV group and the healthy group. The level of BVAS was 25.5 (18.0, 34.0) in the 40 patients with AAV and 0 in the 10 healthy volunteers. The value of Scr was 259.0 (151.5, 422.0) μmol/l, the calculated eGFR was 19.4 (10.7, 42.2) ml/min/1.73 m^2^; renal function was abnormal in 39 out of the 40 patients with AAV. The CRP of each patient in the AAV group was >0.500, while the CRP in the normal group was <0.500 (*P*<0.001). The CRP in the AAV group was 11.4 (2.2, 44.0) mg/l. The D-dimer of AAV patients was 1.5 (0.508, 2.5) μg/ml and of normal volunteers was 0.234 ± 0.093 (range 0.120–0.410) μg/ml. The AAV group and the healthy volunteer group were compared, and one of the groups did not conform to the normal distribution, so the rank-sum test was used (*P*<0.001). PLT in AAV patients was 173.0 (113.0, 212.3) 10^9^/l and in healthy volunteers was 239.9 ± 33.5 10^9^/l. Among the 40 patients with AAV, 4 had cerebral infarction, 2 had myocardial infarction, 4 had cerebral ischemia and 2 had cerebral hemorrhage. The patients with complications of cerebral infarction and myocardial infarction were grouped as thromboembolism. Hence, there were six cases of thromboembolism (15%), four cases of cerebral ischemia (10%) and two cases of cerebral hemorrhage (5%) (Table 1).

**Table 1:** General data of patients with AAV

Parameters	Number
General clinical data	
Subjects, *n*	40
Male/female, *n/n*	19/21
Age at disease onset, means ± SD	57.7 ± 10.7
The level of BVAS, M(P25, P75)	25.5 (18.0, 34.0)
Scr, μmol/l, M(P25, P75)	259.0 (151.5, 422.0)
eGFR, ml/min/1.73m^2^, M(P25, P75)	19.4 (10.7, 42.2
Renal function, *n*	39
CRP, mg/l, M(P25, P75)	11.4 (2.2, 44.0)
D-dimer, μg/ml, M(P25, P75)	1.5 (0.508, 2.5)
PLT, 10^9^/l, M(P25, P75)	1730 (113.0, 212.3)
Cerebral infarction, *n*	4
Myocardial infarction, *n*	2
Cerebral ischemia, *n*	4
Cerebral hemorrhage, *n*	2

### Plasma levels of S1P in AAV were higher than in healthy volunteers

**Figure 1:**
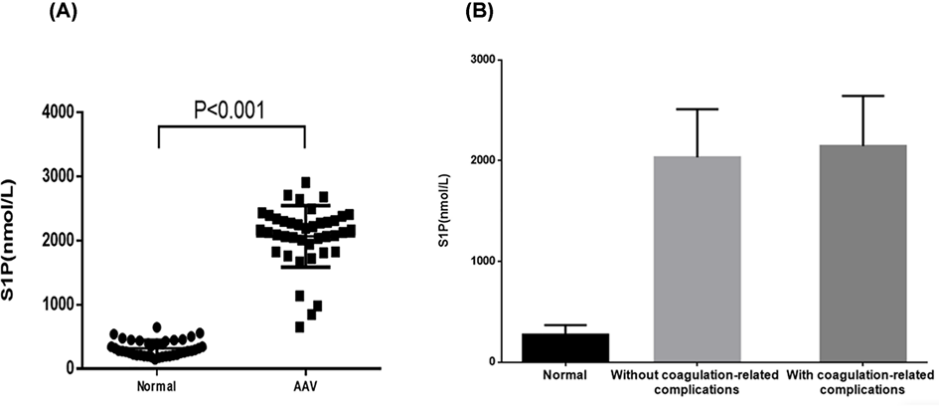
Comparison of plasma S1P levels in healthy volunteers and patients with AAV disease (**A**) Plasma levels of S1P in AAV patients (*n*=40) and healthy volunteers (normal, *n*=10). The two sets of data did not conform to the normal distribution, so the rank-sum test was used (*P*<0.001). (**B**) Plasma levels of S1P were compared among healthy volunteers, AAV patients without coagulation complications and AAV patients with coagulation complications.

In the AAV patients, the plasma S1P level was 2149.7 (1855.9, 2334.5) nmol/l, and in the healthy volunteers, the plasma S1P level was 274.6 ± 94.2 (range 149.2–452.9) nmol/l. Comparison of plasma S1P levels between the two groups showed that the two sets of data did not conform to the normal distribution, so the rank-sum test was used (*P*<0.001) (Figure 1A). We compared the S1P levels of healthy volunteers and AAV patients. The AAV patients were divided into two groups: without and with coagulation-related complications, and no significant differences in the S1P levels was found between the two groups, but both groups showed higher S1P levels than the healthy volunteers (Figure 1B). A previous study found that the AAV patients were in a hypercoagulable state, so we divided the AAV patients with coagulation-related complications into three groups: thromboembolism, cerebral ischemia and cerebral hemorrhage, for further study.

### Plasma S1P levels increased with increase in BVAS and Scr levels, as opposed to eGFR

**Figure 2:**
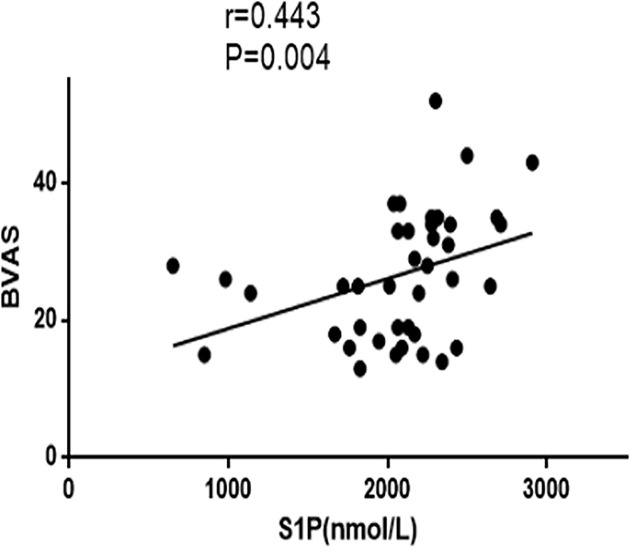
Correlation between plasma levels of S1P and BVAS Plasma S1P levels positively correlated with BVAS (*P*=0.004, *r* = 0.443).

**Figure 3:**
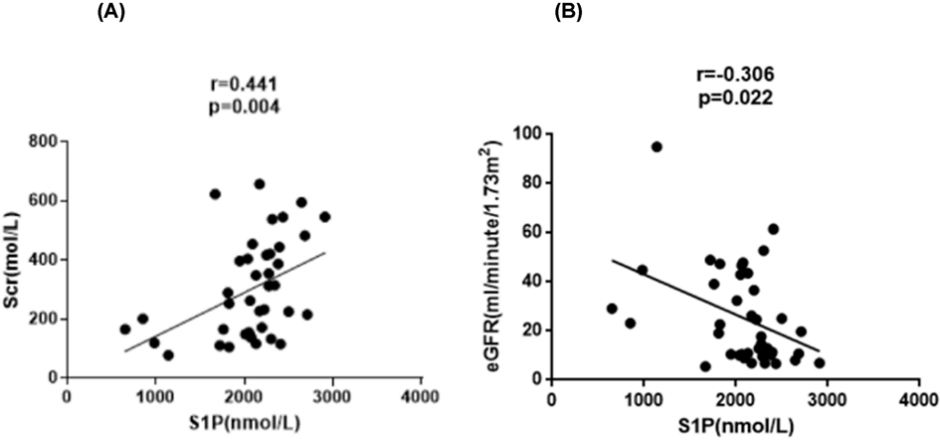
Plasma S1P levels compared with Scr and eGFR, respectively (**A**) The correlation of S1P and Scr. Plasma S1P levels positively correlated with Scr (*P*<0.05, *r* = 0.441). (**B**) The correlation of S1P and eGFR. Plasma S1P levels negatively correlated with eGFR (*P*=0.022, *r* = −0.306).

We analyzed the relationship between the plasma S1P levels and BVAS, CRP, Scr, eGFR in AAV patients. The results showed that plasma S1P levels were positively correlated with BVAS (*r* = 0.443, *P*=0.004, Figure 2); but not with venous blood CRP (*r* = −0.830, *P*=0.609). Moreover, plasma S1P levels were positively correlated with Scr (*r* = 0.441, *P*=0.040, Figure 3A), and negatively correlated with eGFR (*r* = −0.306, *P*=0.022, Figure 3B).

### Coagulation-related clinical indicators: plasma S1P levels were positively correlated with D-dimer and PLT

**Figure 4:**
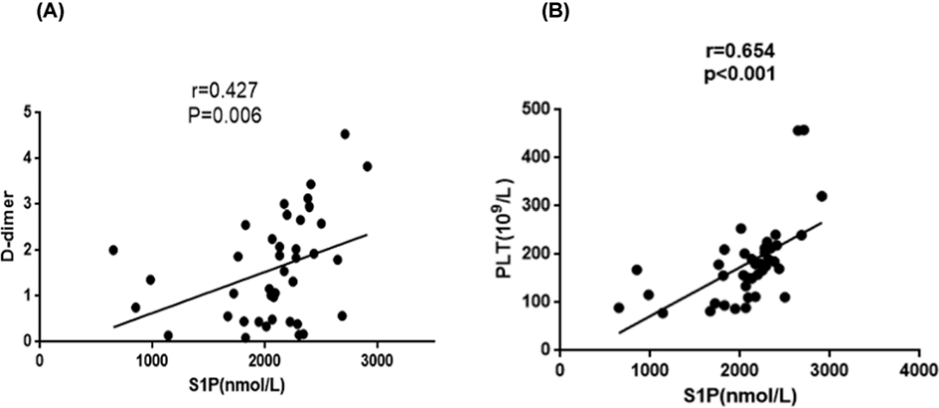
Plasma S1P levels compared with PLT and D-dimer, respectively (**A**) The correlation of S1P and PLT. Plasma S1P levels positively correlated with PLT (P<0.001, *r* = 0.654). (**B**) The correlation of S1P and D-dimer. Plasma S1P levels positively correlated with D-dimer (*P*=0.006, *r* = 0.427).

We compared the plasma levels of S1P with the PT, APTT, TT, FIB, FDP, D-dimer and PLT indicators. There was no correlation between plasma S1P levels and PT, APTT, TT, FIB and FDP, but plasma S1P levels were positively correlated with PLT and D-dimer (*r* = 0.654, *P*<0.001; *r* = 0.427, *P*=0.006, Figure 4A,B) (Table 2).

**Table 2:** Comparison of coagulation-related clinical indicators with S1P

	S1P	
Parameters	*r*	*P*
PT	0.306	0.055
APTT	0.073	0.652
TT	0.101	0.536
FIB	−0.112	0.493
FDP	0.110	0.500
**D-dimer**	**0.427**	**0.006**
**PLT**	**0.654**	**<0.001**

Note: Bold values signify that Plasma S1P is positively correlated with D-dimer and PLT.

### AAV patients with higher plasma levels of S1P were more susceptible to thromboembolisms

**Figure 5:**
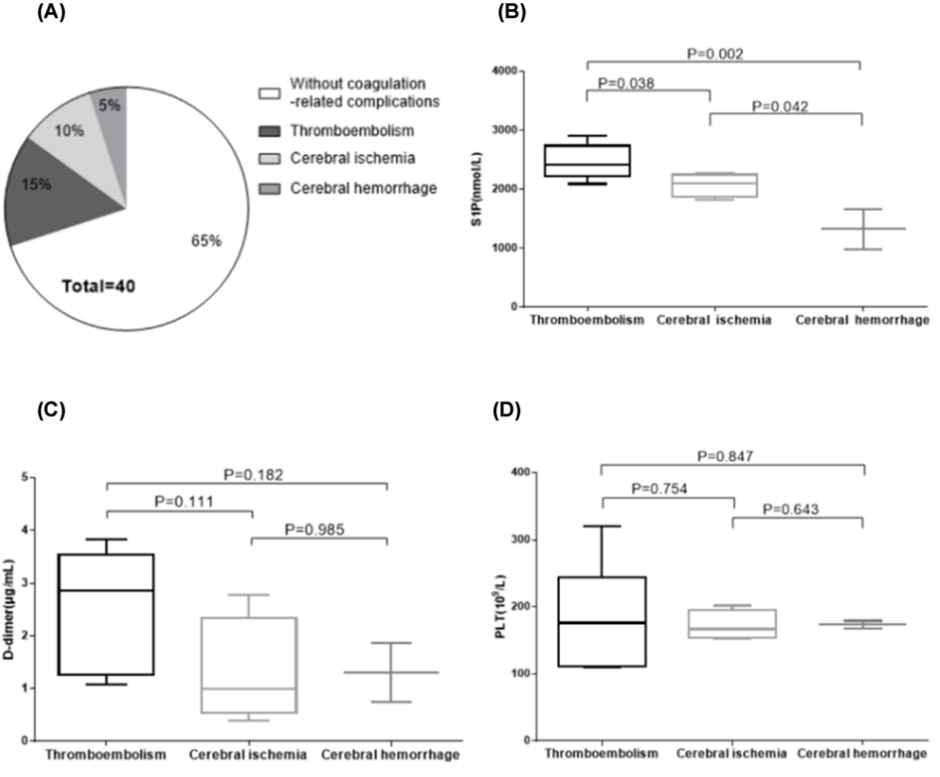
Comparison of the number of coagulation complications and the level of S1P in 40 patients with AAV (**A**) Of the 40 patients with AAV, there was no clinically observed coagulopathy (*n*=28), thromboembolism (*n*=6), cerebral ischemia (*n*=4) and cerebral hemorrhage (*n*=2). (**B**) Among the 40 AAV patients, 12 patients with coagulation complications. There were three groups (thromboembolism, cerebral ischemia, cerebral hemorrhage), for which we compared their S1P levels. S1P levels were highest in the coagulation group and S1P levels were lowest in cerebral hemorrhage (*P*=0.003). (**C**) The complications of coagulation in AAV patients were divided into three groups (thromboembolism, cerebral ischemia, cerebral hemorrhage), and the D-dimer of the three groups was compared. (**D**) The complications of coagulation in AAV patients were divided into three groups (thromboembolism, cerebral ischemia, cerebral hemorrhage), and the D-dimer of the three groups was compared.

When collecting relevant clinical data, we found that 2 of the 40 patients with AAV had a myocardial infarction, 4 had cerebral infarction, 4 had cerebral ischemia and 2 had cerebral hemorrhage, while 28 had no coagulation-related complications. We clubbed the patients with myocardial infarction and cerebral ischemia infarction into the thromboembolism group, so there were six patients with thromboembolic complications. We used s-analysis to compare the coagulation-related complications in the thromboembolism, cerebral ischemia and cerebral hemorrhage groups (Figure 5A). S1P level was the highest in the thromboembolism group, and the lowest in the cerebral hemorrhage group, indicating that higher S1P level increased the patient’s susceptibility to thromboembolism (Figure 5B). We also compared D-dimer and PLT levels in AAV patients with coagulation-related complications (Figure 5C,D), and found that S1P could reflect coagulation-related complications, while PLT and D-dimer could not.

## Discussion

AAV is a chronic disease with a high recurrence rate that affects multiple organs in the body, mainly ear, nose and throat (80%), lung (80%) and kidney (75.4%) [43,44]. Previous studies have shown that plasma S1P levels were higher in the AAV disease group than in the normal group, and were higher in disease activity than in remission stage [9,15]. In this study, the plasma S1P level of AAV patients were detected and BVAS scores were assigned. The results showed that plasma S1P levels were not only higher in AAV patients than in healthy volunteers but also correlated positively with BVAS. Therefore, we hypothesized that plasma S1P levels were related to the disease activity.

In patients with AAV, plasma levels of S1P were significantly increased, which was mainly related to the up-regulation of S1PR2–5 expression in kidneys, and plasma S1P levels were related to kidney expression and disease activity [15]. The increase in plasma S1P level was mainly due to the enhanced expression of S1PR2–5 in the kidneys, and the high expression of S1PR2–5 could promote the activation of glomerular endothelial cells (GenC) mediated by MPO-ANCA-positive IgG [28]. Similarly, increased plasma S1P levels could stimulate the activation of Rho in MPO-ANCA-positive IgG [20]. Therefore, we hypothesized that the level of S1P in plasma may have a pathogenic effect on patients with AAV.

Extensive evidence have shown that S1P can be used as a second messenger to regulate inflammation and immune response [45]. S1P is reported to play an essential role in endogenous neutrophil regulators [46]. Balance of S1PR expression and regulation of Rho and Rac to destroy/maintain the GEnC barrier plays a vital role in the pathogenesis of AAV [20,28,47]. C5a plays a crucial role in the pathogenesis of AAV [9,48–50]. C5a can trigger ANCA-mediated respiratory bursts and degranulation of neutrophils. Moreover, C5a triggers the activation of ANCA, thereby activating the coagulation system and producing thrombin [48,51]. Because S1P is a critical factor in C5a-mediated neutrophil activation, it can potentially play a role in the inflammatory and coagulation systems [48,52]. S1P interacts with C5a to cause ANCA-induced neutrophil respiratory bursts and degradation in AAV. Previous studies found that the complement system plays a vital role in the pathogenesis of AAV in mouse models and humans [9,22,53,54]. Among them, C5a plays a crucial role in ANCA-mediated neutrophil activation and recruitment [25].

C5a and ANCA stimulation of neutrophils not only causes neutrophil respiratory bursts and degranulation but also activates the coagulation system and produces thrombin. In a previous study of 46 AAV patients, 30 kidney biopsies were performed of which eight samples showed histological evidence of thrombotic microangiopathy (TMA) in the kidneys [55,56]. Because S1P may up-regulate the expression of C5aR on the surface of neutrophils and activate C5a, S1P release and activated C5a can induce tissues to express coagulation factors from neutrophils and endothelial cells, thereby triggering the exogenous coagulation system [56,57]. PLT and the complement system are components of innate immunity. PLT is activated in patients with AAV, and this activation is also related to the thrombin pathway [56,58,59].

PAR1 is a G-protein coupled family receptor. PAR1 can activate thrombin to enhance the activation of GEnC induced by MPO-ANCA-positive IgG. S1P can induce the expression of PAR1 in GEnCs treated with MP1-ANCA-positive IgG, which can enhance the appearance of tissue factor (TF) and promote coagulation, and further activate the coagulation system, to initiate a vicious circle [48]. Patients with AAV were found to be in a hypercoagulable state [43]. Thrombin can activate the SphK1-S1P-S1PR3 axis and promote S1P production. Synergistic effects of thrombin and SphK-S1P-S1PR3 signal transduction leads to endothelial barrier dysfunction [20,48]. Given the hypercoagulable state of AAV patients, they have increased venous thromboembolism [29]. Therefore, we believed that the level of plasma S1P is related to the hypercoagulability in AAV patients. However, the relationship between plasma S1P levels and coagulation-related indicators has not been previously analyzed. Therefore, we examined the level of plasma S1P and analyzed its relationship with the clinical indicators of abnormal blood coagulation in AAV patients, and also examined the relationship with BVAS.

In the present study, plasma levels of S1P in patients with AAV were found to be significantly higher than those in healthy volunteers. The correlation analysis showed that in patients with AAV, plasma level of S1P was positively correlated with Scr and negatively correlated with eGFR. Therefore, we hypothesized that the plasma levels of S1P may reflect the degree of impaired renal function. Moreover, the plasma levels of S1P in AAV patients was positively correlated with D-dimer, BVAS and PLT. We compared S1P, PLT and D-dimer in AAV patients with coagulation complications, and found that S1P was specific in coagulation complications, but PLT and D-dimer were not (Figure 5). Therefore, we believed that S1P can be used as a biomarker for coagulation-related complications in AAV patients.

## Conclusion

Plasma S1P can be used as a biomarker in AAV since the levels of S1P could predict thromboembolic complications in AAV patients.

## Abbreviations

AAV: ANCA-associated vasculitis
ANCA: anti-neutrophil cytoplasmic antibody
APTT: partial thromboplastin time
BVAS: Birmingham vasculitis activity score
CRP: C-reactive protein
eGFR: estimated glomerular filtration rate
FDP: fibrinogen reduction product
FIB: fibrinogen
GBM: Anti-basement membrane antibody glomerulonephritis
MDRD: Modification of Diet in Renal Disease
MPO: myeloperoxidase
PLT: platelet
PR3: protease 3
PT: prothrombin time
Scr: serum creatinine
SK: sphingosine kinase
SLE: systemic lupus erythematosus
S1P: sphingosine-1-phosphate
TNF-α: tumor necrosis factor-α
TT: thrombin time
